# Error Injection in Task-Based Image Quality Pipelines: What Regression Testing Cannot Catch, and Why Neither Internal Identities nor Closed-Form References Suffice Alone

**DOI:** 10.3390/jimaging12090443

**Published:** 2026-09-14

**Authors:** Shuji Yamamoto

**Affiliations:** Institute of One, LISIT Co., Ltd., Tokyo 150-0044, Japan; yamamoto@lisit.jp

**Keywords:** task-based image quality, model observer, MTF, NPS, NEQ, detectability, error injection, implementation error, quality assurance, closed-form validation

## Abstract

Task-based image quality assessment—the modulation transfer function, the noise power spectrum (NPS), the noise-equivalent quanta and model observers—fails by returning a plausible wrong number rather than an error, and the regression test most implementations carry cannot tell a plausible right answer from a plausible wrong one, because the stored reference was recorded from the defective code. We injected six defects into a validated implementation of that chain through a severity dial that recovers the correct pipeline exactly at zero. A self-consistency regression test detected none of the six. Four internal identities, which need no ground truth, and two closed-form references, which need a phantom whose answer is known, together detected all six, in every case at or before the severity at which the reported detectability index $d^{\prime }$ became wrong by more than 5%—an error that three of the six never produced. Neither family sufficed alone: the internal identities detected three of the six and the closed-form references five, and peak errors in a reported $d^{\prime }$ reached 90%. Run unmodified on measured American College of Radiology (ACR) phantom projections across seven reconstruction kernels, the three identities that can be evaluated without ground truth transferred intact—Parseval held to $4\times 10^{-16}$—but their tolerances did not, and the strongest apodisation drove the noise dynamic range to within 0.6% of the threshold beyond which a prewhitening observer should return no value at all rather than a computed one, because 1/NPS has ceased to be numerically meaningful.

## 1. Introduction

Task-based assessment—judging an imaging system by how well a specified observer performs a specified detection or discrimination task, rather than by a generic fidelity metric—is the accepted framework for evaluating medical imaging systems [1,2]. Its physical ingredients are individually standardised: the modulation transfer function (MTF), classically measured from a slanted edge by the presampled-MTF method formalised by the International Organization for Standardization (ISO) in ISO 12233 [3], and the noise power spectrum (NPS) and detective-quantum-efficiency formalism standardised for digital X-ray detectors by the International Electrotechnical Commission (IEC) in IEC 62220-1 [4]. Its observer theory is mature [1,5,6,7], its relation to dose and patient risk has been set out in detail [8], its practice from physical measurement through to model observers has been reviewed for computed tomography (CT) [9], and its use in CT has been consolidated in American Association of Physicists in Medicine (AAPM) Task Group 233 [10]. The quantity tying the physics to the task is the noise-equivalent quanta (NEQ), $\mathrm{NEQ}={\mathrm{MTF}}^{2}/\mathrm{NPS}$, because the ideal-observer detectability of a known signal imaged through a linear system is exactly an integral of NEQ against the signal’s power spectrum.

*Detectability* here is the index $d^{\prime }$, the separation between the observer’s response to signal-present and signal-absent images divided by the standard deviation of that response. It is dimensionless: $d^{\prime }=1$ means the two distributions are one standard deviation apart, and $d^{\prime }\approx 2$ corresponds to roughly 92% correct in a two-alternative forced choice. For the ideal prewhitening observer, it is computed in closed form from the signal and the noise spectrum rather than by simulating decisions.

A *phantom* is an object of known composition imaged in place of a patient; a *synthetic phantom* is one that exists only as an array of numbers, generated rather than scanned, so that the answer the estimator should return is known analytically. The distinction matters throughout this paper: a synthetic phantom supplies ground truth, and a physical one does not.

The theory is settled. The implementations are not, and this paper is about the gap between them. The object of study is therefore the chain itself—any implementation of it—rather than a particular programme: each defect examined below is a step the standard formulation requires, applied to one instance so that its cost can be measured, and the checks proposed against them are stated so that they can be asserted inside any implementation.

### 1.1. The Characteristic Failure Is a Plausible Number

A pipeline assembling this chain fails in a specific and inconvenient way. It does not crash, and it does not return an obviously absurd value. It returns a number of the right order of magnitude, with the right units, moving in the right direction when the dose is changed—and wrong.

Three concrete examples follow, all found in the pipeline studied here during its development:

- A slanted-edge MTF routine locates each edge-profile sample in the bin it falls into, but uses the bin *centre* as its position. The mean sample position inside a bin is not the bin centre, and the resulting position jitter biases the estimate. The bias depends on the edge angle, so it is invisible to anyone who tests at one angle.
- A prewhitening observer weights by 1/NPS. Handed a noise model whose power decays below floating-point underflow—that is, where the computed NPS falls below the smallest number the arithmetic can represent and is stored as a denormal or as zero—it weights those bins by 1/NPS and so multiplies them by an enormous factor. The resulting $d^{\prime }$ is of order $10^{29}$: not a large detectability but an arithmetic artefact, assembled entirely from bins where the “signal” is rounding error. A plausible $d^{\prime }$ is a number near unity, so this one is at least visibly wrong; the same mechanism at milder decay produces one that is not.
- A soft-edged disc phantom, blurred by applying a one-dimensional edge profile radially rather than by an exact two-dimensional convolution, gains $\pi \sigma^{2}$ of area. The detectable signal energy then depends on the blur without any indication that it does, so every conclusion drawn about resolution is contaminated by a change in the signal itself.

None of these announce themselves. Each produces a number a reviewer would accept.

### 1.2. What a Regression Test Can and Cannot Establish

The standard defence is a regression test: run the pipeline, store the output and fail the build if the output ever changes. This is a genuinely useful discipline—it catches accidental change, and it makes refactoring safe. It also cannot, in principle, catch any of the three defects above.

The reason is structural rather than incidental. If a defect was present when the reference output was recorded—which is the normal case for a defect that was never noticed—then the stored value *is* the defective value. The pipeline is deterministic, so it reproduces that value exactly, and the test passes. A regression test establishes that the code is stable. Stability and correctness are different properties, and the reproducibility apparatus of computational science largely certifies the first [11]: an analysis that re-runs to the same answer has been shown to be deterministic, not to be right. That software defects reach published results, and are rarely found by the practices meant to prevent them, is documented across fields [12,13].

What can catch such defects is a check whose reference comes from outside the code. Two kinds are available, and the distinction between them turns out to matter:

- **Internal identities** must hold for algebraic reasons, whatever the data. That the integral of the NPS over the frequency plane equals the pixel variance is not an empirical fact about a particular phantom; it is Parseval’s theorem. Identities of this kind need no ground truth and therefore continue to work on measured patient data where no truth exists.
- Neither idea is new to software engineering, and it is worth naming what they are. A check that must hold whatever the input is a *property* in the sense of property-based testing [14], where properties are asserted over generated inputs rather than over stored examples; the internal identities here are properties of that kind, with the algebra of the imaging chain supplying the property instead of a programmer’s intuition. A check comparing two routes to the same quantity, or the same computation under a transformation that should leave the answer predictable, is a *metamorphic relation* [15], the standard response to the oracle problem—the situation, exactly this paper’s situation, where correct output cannot be recognised by inspection. Verification practice for computational science has made the same argument from the scientific side [16].
- What this paper adds is not the idea but its content for one chain: which relations exist in task-based image quality, what each detects, what it costs, and—the part that cannot be borrowed—the finding that the two families are not interchangeable.
- **Closed-form references** compare an estimate to the analytic answer for an object constructed so that the answer is known: an analytically blurred edge whose presampled MTF is exactly $\exp\left(-2\pi^{2}\sigma^{2}f^{2}\right)$, or white noise of known variance whose spectrum is exactly $\sigma^{2}\;\Delta x\;\Delta y$. These are stronger, and they require a phantom.

Whether the second kind is worth its cost—whether a synthetic phantom earns its place in a workflow whose object is real images—is an empirical question that this paper answers.

### 1.3. Contributions

1. A controlled **injection study**: six defects, each dialled by a severity that recovers the correct pipeline exactly at zero, evaluated against a self-consistency regression test and six checks, with the severity of first detection compared against the severity at which the reported $d^{\prime }$ first becomes materially wrong.
2. The finding that the two check families are **complementary and individually insufficient**, and in particular, that half of these defects are undetectable without a phantom of known truth.
3. Two **corrected magnitudes** for defects previously reported only qualitatively: the angular structure of the bin-centre jitter bias and the dependence of the omitted-sinc bias on a free implementation parameter.
4. A demonstration that the same chain, unmodified, reproduces the required behaviour on **measured projections from a clinical scanner** across a seven-point reconstruction-kernel sweep.

## 2. Materials and Methods

### 2.1. The Pipeline Under Test

All experiments use one pure-Python implementation of the chain (taskiq-core v0.4.0), which computes the slanted-edge MTF, the two-dimensional and radially averaged NPS, the unnormalised $\mathrm{NEQ}={\mathrm{MTF}}^{2}/\mathrm{NPS}$, and model-observer detectability. Observers are the non-prewhitening matched filter with an optional eye filter (NPW/NPWE) [17], the prewhitening matched filter, which coincides with the ideal linear observer for stationary Gaussian noise, and the channelised Hotelling observer [5]. Task performance is summarised by $d^{\prime }$, the area under the receiver operating characteristic (ROC) curve computed distribution-free from the Mann–Whitney statistic, and two-alternative-forced-choice percent correct.

The MTF estimator forms the edge-spread function—the ESF, the mean profile across the edge obtained by projecting every pixel onto the edge normal and binning far below the pixel pitch, which is what the edge’s tilt buys—differentiates it to the line-spread function, the LSF, by a central difference between adjacent bins, and transforms the LSF, analytically dividing out the two transfer functions the estimator itself introduces: the bin-average boxcar, sinc(fh), and the central-difference derivative, sinc(2fh), where h is the ESF bin width. It additionally corrects each bin from its measured mean sample position to the bin centre. The NPS estimator detrends each region of interest and normalises so that the integral of the NPS over the frequency plane equals the pixel variance as an exact identity.

Unless stated otherwise the reference configuration is pixel pitch Δx=0.1 mm, system blur σ=0.15 mm, a 256×256 edge phantom at 5°, a 64×64 noise region over 64 realisations at noise standard deviation 20 units, and a disc signal of radius 0.8 mm and contrast 6 units. The reference pipeline returns $d^{\prime }=3.5745$ from the prewhitening observer and $d^{\prime }=3.5719$ through the NEQ route.

### 2.2. The Checks

Six checks were implemented: four internal identities and two closed-form references (Table 1). Each returns a scalar violation magnitude; a check *fires* when that magnitude exceeds its tolerance.

Tolerances are not chosen for convenience. Each is set from the estimator’s own measured reproducibility on the correct pipeline, with an order of magnitude of margin: the residual of mtf_closed_form on a correct run is $4.9\times 10^{-6}$, so its tolerance is $5\times 10^{-5}$; the residual of nps_closed_form is $3.6\times 10^{-2}$ against a tolerance of 0.25; parseval and bridge hold to $2.2\times 10^{-16}$ and exactly zero, respectively. A tolerance set tighter than the estimator’s reproducibility produces a check that fires on correct code, which is not a sensitive guard but a broken one—a failure mode we encountered and discuss in Section 3.6.

Two points deserve emphasis. First, the bridge check states that the two routes to ideal-observer detectability agree:

$${\left(d^{\prime }\right)}_{\mathrm{NEQ}}^{2}=\int \mathrm{NEQ}\left(f\right){\left|S\left(f\right)\right|}^{2}df\;\;\;\mathrm{and}\;\;{\left(d^{\prime }\right)}_{\mathrm{PW}}^{2}=\frac{\int {\left|S\left(f\right)\right|}^{2}}{\mathrm{NPS}\left(f\right){\left|\mathrm{MTF}\left(f\right)\right|}^{2}df}$$

where S(f) is the Fourier transform of the signal, and the check is the relative difference $\left|{\left(d^{\prime }\right)}_{\mathrm{NEQ}}^{2}-{\left(d^{\prime }\right)}_{\mathrm{PW}}^{2}\right|/{\left(d^{\prime }\right)}_{\mathrm{PW}}^{2}$. With $\mathrm{NEQ}={\mathrm{MTF}}^{2/\mathrm{NPS}}$ the two integrands are the same expression rearranged, which is why the check is an identity rather than an approximation. It compares two routes to the same number—the prewhitening observer applied to the imaged signal, and the integral of NEQ against the object’s power spectrum—that share no code path but are, under the discrete conventions used here, the same integral rearranged. It is therefore an exact identity rather than an approximation and holds to zero on the reference pipeline. It is only an identity when both routes use the same noise model; comparing routes that disagree about the noise tests nothing but that disagreement. Second, dynamic_range excludes the zero-frequency (DC) bin. Subtracting the mean from each region of interest (ROI) sets the zero-frequency component to zero up to rounding, so the DC bin holds a value of order $10^{-31}$—the residue of that subtraction, not a measurement. Including it would make the ratio of largest to smallest NPS value enormous for *any* field, white noise included, and the check would fire on correct code every time. The exclusion is bookkeeping about how the estimator works, not a claim about the spectrum.

### 2.3. The Self-Consistency Control

The control is the test most implementations actually have. The pipeline is run at a given severity, its output recorded, and the pipeline re-run and compared to that record. Critically, the record is taken from the pipeline *as it stands*, with the defect already present—the realistic case for a defect that was never noticed. The pipeline is deterministic, so the comparison is exact by construction. Reporting this as a result rather than asserting it is deliberate: it is the quantitative form of the argument in Section 1.2.

### 2.4. The Six Defects

Each defect is applied to the correct pipeline through a severity α∈[0,1] constructed so that α=0 recovers the correct pipeline exactly. Severity is an exact parameter of the injection, not an estimate of anything: it is the interpolation weight between the correct implementation and the defective one, defined separately for each defect in the list below and computed rather than measured. A *severity dial* is that parameter used as a continuous control, which is what makes the experiment possible—a defect that can only be present or absent gives one data point, whereas one that can be turned up from nothing gives a curve, and the curve is where the comparison between detection and materiality lives. Nothing is rewritten to break it; the defect is injected from outside the library.

1. **sinc**—the bin-average and central-difference transfer functions left in the MTF estimate, at severity α: ${\mathrm{MTF}}_{\alpha }=\mathrm{MTF}\cdot {\left[\mathrm{sinc}\left(fh\right)\;\mathrm{sinc}\left(2fh\right)\right]}^{\alpha }$. Evaluated at an ESF bin of Δx/4
2. **jitter**—the bin-centre correction omitted, with severity indexing the edge angle over eleven values from 2° to 20°.
3. **nps_area**—the pixel-area factor dropped from the NPS normalisation by a fraction α: ${\mathrm{NPS}}_{\alpha }=\mathrm{NPS}\cdot {\left(\Delta x\;\Delta y\right)}^{-\alpha }$
4. **detrend**—ROI detrending switched off over a background ramp of amplitude 60α units.
5. **disk—the signal built** by interpolating between the exact two-dimensional blurred disc and the one obtained by applying the one-dimensional edge profile radially.
6. **no_floor**—the prewhitening observer handed a measured spectrum with noise correlation length 0.5α mm and no floor.

Defects 2, 5 and 6 are the three found in this pipeline during development; 1, 3 and 4 are standard failure modes of the same chain.

### 2.5. Detection and Materiality

For each defect the *detection severity* is the smallest α at which any check fires, and the *material severity* is the smallest α>0 at which the reported $d^{\prime }$—taken as the worse of the prewhitening and NEQ routes—differs from that defect’s own α=0 value by more than 5%. Measuring against the defect’s own zero rather than a global reference removes confounds: the jitter sweep varies the edge angle, which changes the estimate slightly even when the correction is applied.

A check that fires only after the answer is already wrong is not a guard, so the comparison of these two severities, and not the mere fact of detection, is the endpoint.

**The grid, and what it can and cannot resolve.** Every defect is evaluated on the same eleven-point grid, α∈{0,0.1,0.2,…,1.0}, with 64 noise realisations at each point and the random streams fixed by seed so that a rerun reproduces the table exactly. Seven of the eight detections in Table 2 occur at α=0.1, which is the smallest non-zero severity examined. **That is a property of the grid as much as of the checks**: these checks fire at or below a tenth of full severity, and this experiment cannot say how much below, because it never looked. The claim made here is therefore that detection occurs at or before materiality—for nps_area and no_floor the two fall on the same grid point—which the grid does resolve, and not that any particular detection threshold has been measured, which it does not.

Each tolerance is set from the estimator’s own reproducibility on the correct pipeline rather than chosen to make a check succeed: the measured residual over the 64 realisations is taken as the noise floor and the tolerance placed an order of magnitude above it, as listed in Table 1. A tolerance below that floor yields a check that fires on correct code, which is discussed in Section 3.6.

### 2.6. The Real-Scanner Arm

To establish that the chain behaves as required outside a synthetic model, the same code was run on measured American College of Radiology (ACR) phantom projections from LDCT-and-Projection-data [18], acquired on a Siemens CT scanner (Siemens Healthineers, Forchheim, Germany). The ACR accreditation phantom is an established vehicle for measuring MTF and NPS on a clinical scanner [19], which is why it was chosen (The Cancer Imaging Archive, CC BY 4.0). Nothing about the acquisition is simulated: one set of measured projections was reconstructed seven times with progressively stronger apodisation—a bare ramp, then Hann windows at cutoffs 1.00, 0.80, 0.60, 0.45, 0.35 and 0.25—which moves the MTF and the NPS together exactly as changing a scanner’s reconstruction kernel does. The MTF, NPS, NEQ and model-observer detectabilities were then read off with the same estimators used throughout the study.

### 2.7. Implementation

The estimators, the phantoms and the observers are taskiq-core, a pure-Python package depending only on NumPy and SciPy, with Matplotlib for figures; the results reported here were produced with Python 3.14.3, NumPy 2.5.1, SciPy 1.17.1 and Matplotlib 3.10.8. It contains no Digital Imaging and Communications in Medicine (DICOM) handling and no patient data by design, which is what allows every quantity in it to be checked against a closed form. The MTF, NPS, NEQ and observer routines are the ones named in Section 2.2; the injection study is a single script, paper/make_injection_study.py, that imports them unmodified and applies each defect through the severity dial rather than by editing the library. Reconstruction from real projection data is not in that package but in ldct-io, for the same reason: the archive, its private tags and its geometry have no business inside a library whose correctness is established analytically. The test suite runs on Python 3.10 to 3.12 in continuous integration, and asserts the identities of Section 4.2 on the correct pipeline as well as the study’s own preconditions.

### 2.8. Use of Generative Artificial Intelligence (AI)

A large language model (Claude Opus 5, Anthropic) was used as a tool. Concretely: it drafted initial versions of the estimator and phantom routines and of the test suite, wrote the figure and study scripts, refactored code the author had written, and drafted and edited manuscript text. It did not choose which defects to inject, did not set any tolerance, and did not decide what the results mean.

The relationship between that assistance and this paper’s subject is not incidental and is stated plainly here. Three of the six defects studied were not invented for the experiment: they were real errors present in drafted code—the bin-centre position error, the radially blurred disc, and the missing noise floor—found by the closed-form checks described here and only then turned into a controlled injection. The checks were the means of verifying the assistance, which is the argument of the paper applied to its own production. The author independently re-executed every numerical result reported here and verified all figures, equations and claims against the code. No AI system is an author.

## 3. Results

### 3.1. The Pipeline Is Correct Where a Closed Form Can Reach It

**Figure 1:**
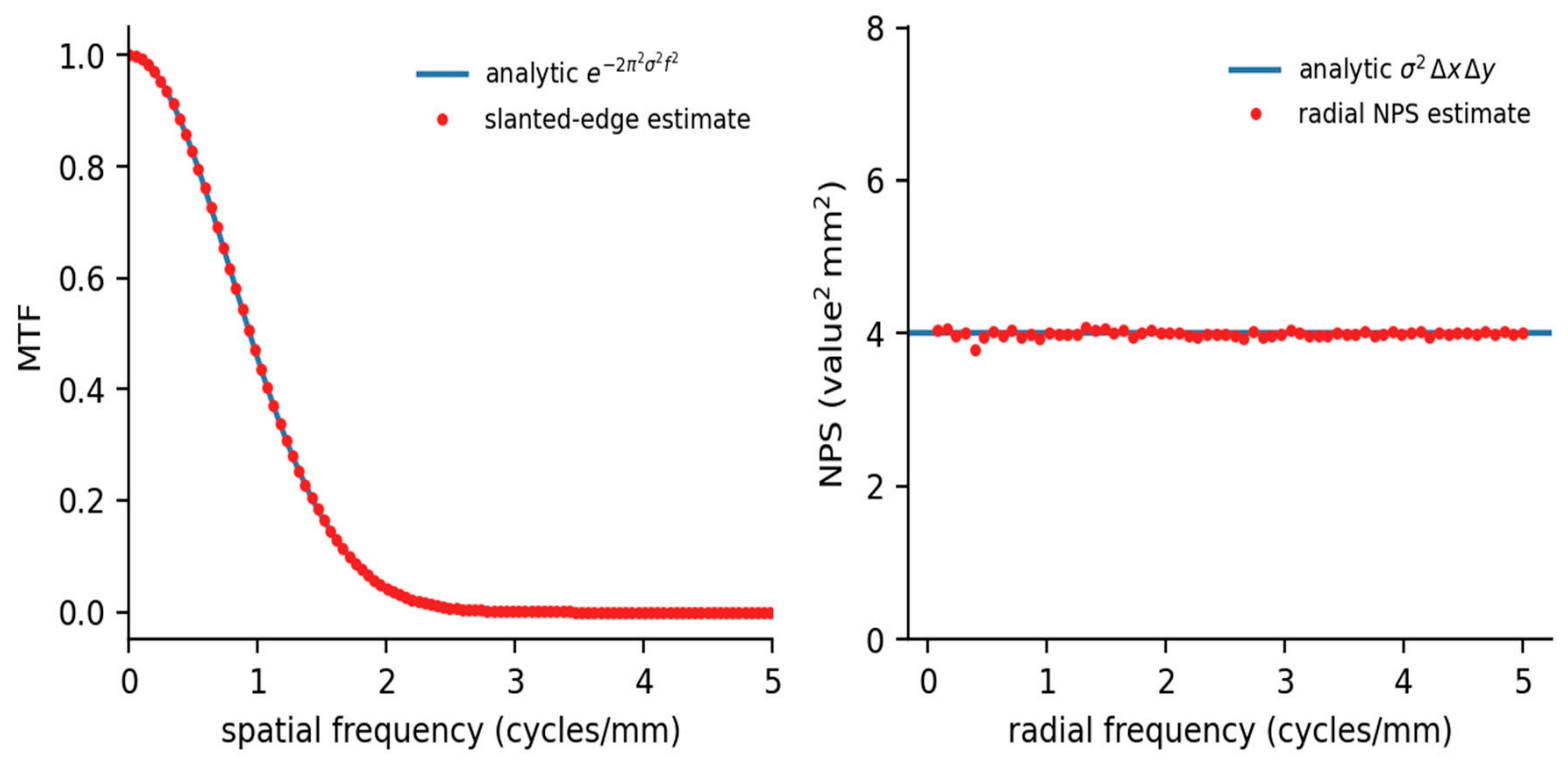
The physical estimators held to their closed forms. Left: the slanted-edge MTF estimate (points) against the analytic Gaussian $\exp\left(-2\pi^{2}\sigma^{2}f^{2}\right)$ (line) for an edge of blur 0.2 mm. Right: the radially averaged NPS estimate (points) against the analytic white level $\sigma^{2}\;\Delta x\;\Delta y$ (line) for noise of standard deviation 20 units.

**Figure 2:**
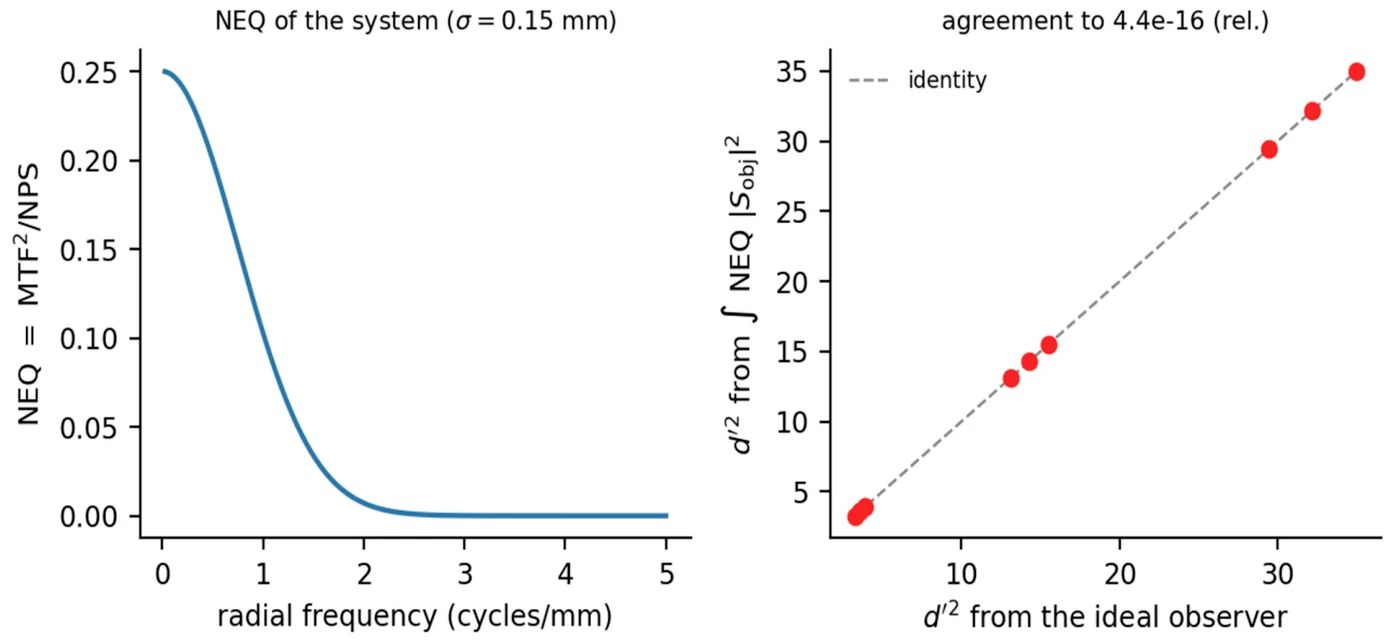
The physics-to-task bridge. Left: the $\mathrm{NEQ}={\mathrm{MTF}}^{2}/\mathrm{NPS}$ of the system. Right: $d^{\prime 2}$ from the prewhitening observer (horizontal) against $d^{\prime 2}$ from integrating NEQ against the object power spectrum (vertical), over nine contrast-by-blur conditions; the points lie on the identity line to machine precision.

**Figure 3:**
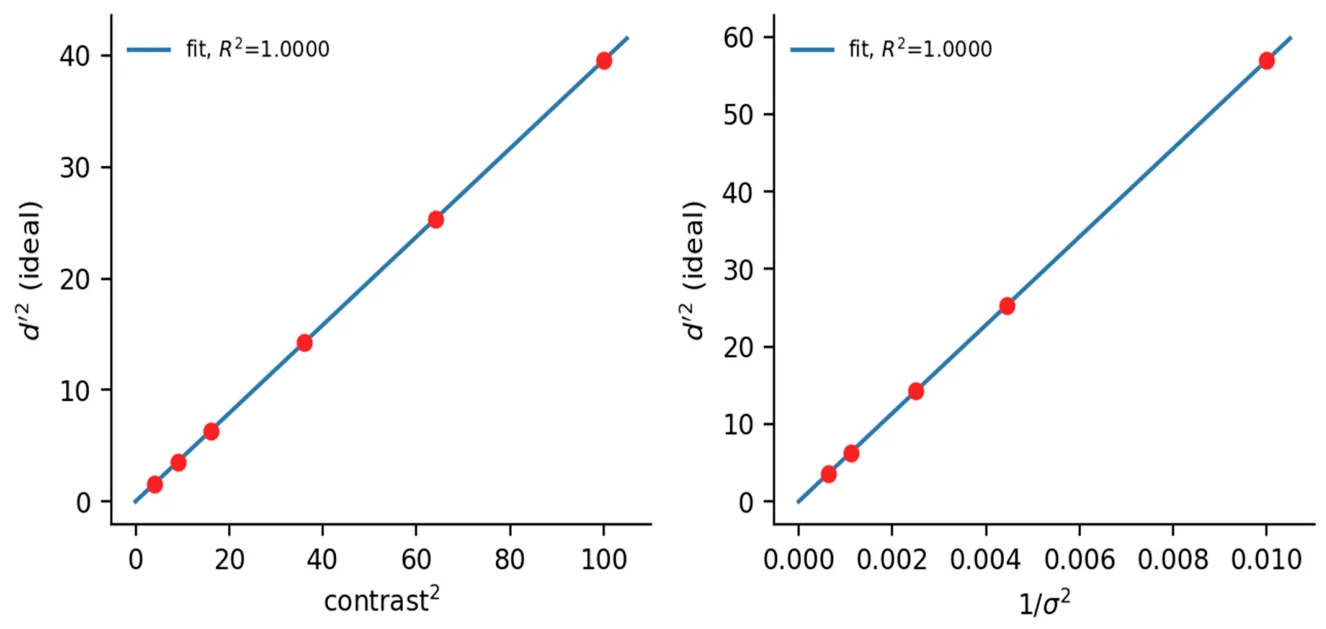
The transfer laws recovered from swept data. Left: ideal-observer $d^{\prime 2}$ against contrast ^2^. Right: $d^{\prime 2}$ against inverse noise variance. Both through-origin fits return a coefficient of determination indistinguishable from 1.

Before injecting anything, the estimators were held to their analytic answers. The slanted-edge MTF matched $\exp\left(-2\pi^{2}\sigma^{2}f^{2}\right)$ to a maximum relative error of **0.004%** (Figure 1) across fifteen blur-by-angle combinations (blur 0.15–0.35 mm, angles 3–15°). Integrating the estimated two-dimensional NPS over the frequency plane recovered the input variance to **0.029%** over 128 realisations—a sampling error, not a bias—with the underlying Parseval identity verified to $6.7\times 10^{-5}$ in absolute residual and to $10^{-10}$ relative in the test suite. The NEQ route and the prewhitening observer agreed to $4.4\times 10^{-16}$ across nine contrast-by-blur conditions (Figure 2). On swept data (Figure 3), $d^{\prime 2}$ was linear in contrast ^2^ and in inverse noise variance with coefficients of determination numerically equal to 1, and the NPWE observer’s efficiency relative to the ideal observer was constant across contrast to a spread of $3.4\times 10^{-9}$.

These establish that the pipeline is correct in the region a closed form can reach. They are the precondition for the injection study, not its result.

### 3.2. Which Check Catches Which Defect

**Figure 4:**
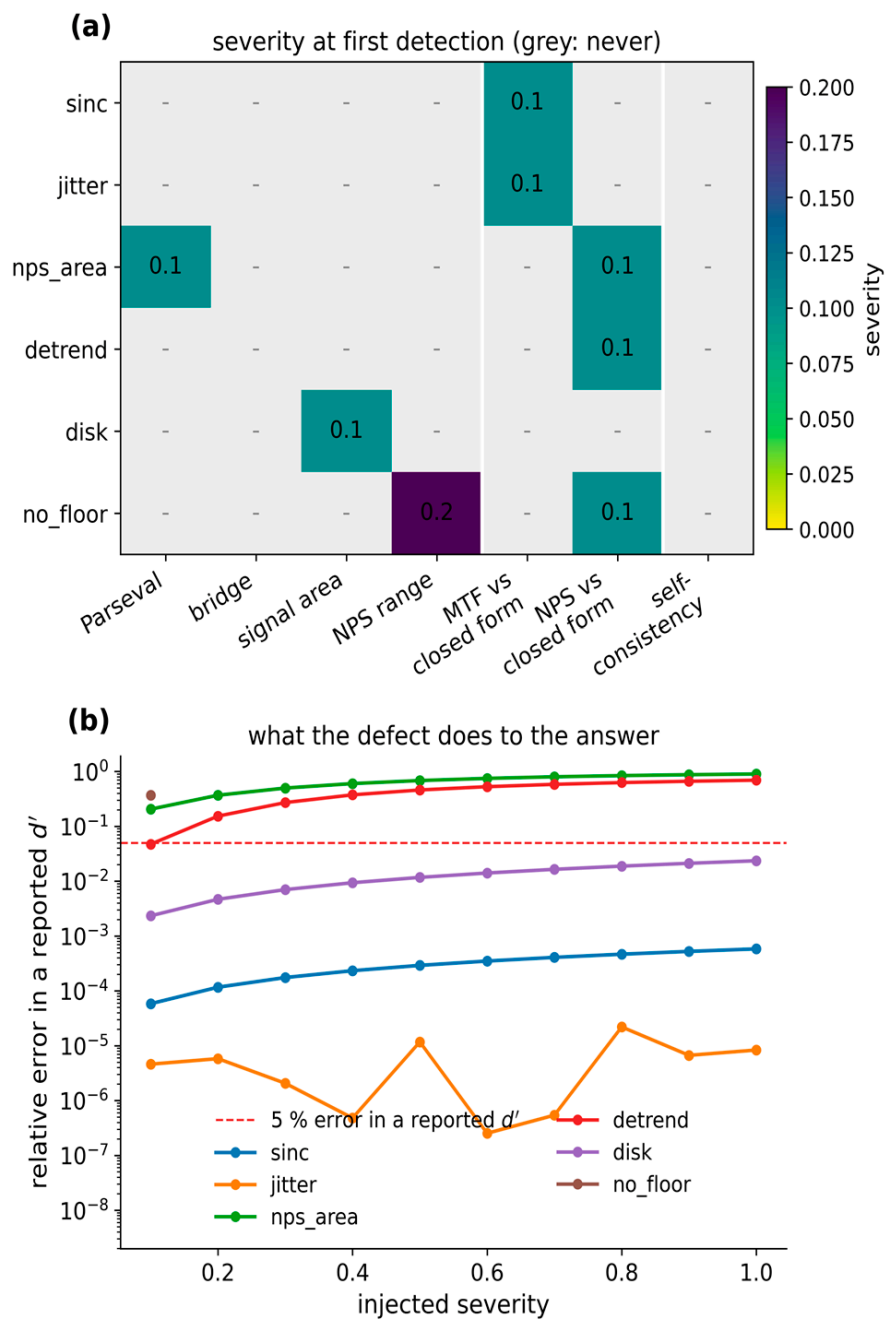
What each check sees. (**a**) The injected severity at which each check first fires, printed in the cell and shown by colour, grey where the check never fires; the rightmost column is the self-consistency regression test, grey for every defect. The white rules separate internal identities (left) from closed-form references (centre) and from the regression control (right). (**b**) The relative error each defect produces in a reported $d^{\prime }$, against the 5% materiality threshold (dashed).

Table 2 and Figure 4 give the outcome. No check fires at α=0 for any defect.

Every defect was caught, and every defect was caught at or before the severity at which it corrupted the answer. Three of the six never made $d^{\prime }$ materially wrong at any severity tried, and were nonetheless detected at the first severity step—which is the desired asymmetry: the checks are more sensitive than the endpoint they protect. 

### 3.3. The Regression Test Catches Nothing

The self-consistency control disagreed with itself by at most 0.0—exactly zero, at every severity of every defect. It caught **0 of 6**.

This is not a subtle result and it is not a criticism of any particular codebase. It follows from determinism: the snapshot was recorded from the defective pipeline, so the defective pipeline reproduces it. Any project whose validation consists of comparing today’s output to yesterday’s has, with respect to defects of this class, no validation at all.

### 3.4. Neither Family of Check Is Sufficient

The two families overlap, and neither covers the whole set:

- **Internal identities** caught nps_area (Parseval), disk (conserved signal area) and no_floor (NPS dynamic range)—**3 of 6**.
- **Closed-form references caught sinc, jitter and detrend, and also nps_area and no_floor—5 of 6.**

Two defects, nps_area and no_floor, were caught by both families; disk was caught by an identity alone, and sinc, jitter and detrend by a closed-form reference alone. No single check caught more than three defects, and only the NPS closed-form reference caught that many. The bridge identity, which is the most theoretically satisfying of the six and holds to machine precision, caught none of these defects at all: it is insensitive to any error shared by both of its routes.

The practical consequence is the paper’s main claim. Internal identities are the checks that survive contact with measured data, because they need no truth; they are what remains available when the object of study is a patient image. They catch half of these defects. The other half—an MTF biassed by its own estimator, an NPS whose level is wrong—are detectable only against an object whose answer is known in advance. Removing the synthetic phantom from a validated workflow does not merely reduce coverage; it removes an entire failure class from view.

### 3.5. Two Magnitudes, Measured

Two defects previously described only qualitatively were quantified, and both descriptions needed revision.

**The jitter bias spikes; it does not drift.** Sweeping the edge angle from 2° to 20° in 0.5° steps with the bin-centre correction disabled, the maximum absolute MTF error over the band f≤3 cycles/mm was $3.2\times 10^{-3}$ at 5.0° but only $8.4\times 10^{-5}$ at 4.5° and $2.8\times 10^{-4}$ at 5.5°—a factor of thirty-eight between neighbouring half-degree steps. The worst case over the whole range is 0.32%, not the 1.4% previously reported for this estimator, and the more important correction is structural: the bias is not a smooth function of angle that one could bound by testing the endpoints. It spikes where the sampling geometry becomes commensurate. With the correction applied the same sweep stays below $5\times 10^{-6}$ throughout.

**The sinc bias is set by a free parameter.** The magnitude of an omitted sinc(fh) sinc(2fh) deconvolution depends entirely on the ESF bin width h, which is an implementation choice rather than a property of the imaging system. Over the same band the deficit is 16.7% at h=Δx/2, 4.3% at Δx/4, 0.70% at Δx/10 and 0.18% at Δx/20. A statement that neglecting these corrections costs “a few percent” is therefore incomplete without the binning: two implementations with the same bug can differ by two orders of magnitude in how much it costs them.

### 3.6. Three Ways to Make a Check Lie

The study as first written reported that every defect was detected at severity zero—an apparently excellent result, and entirely an artefact. Three independent causes were responsible, each of which produced output that read as a finding:

1. The DC bin of a mean-detrended NPS sits at $∼10^{-31}$ by construction. Read as a decayed noise spectrum, it gives a dynamic range of $2\times 10^{31}$ and fires the dynamic-range check on every field, white noise included.
2. The bridge check compared a prewhitening observer using a measured spectrum against an NEQ route using an analytic scalar. The two disagree by $7.7\times 10^{-3}$ for reasons that have nothing to do with any defect.
3. The mtf_closed_form tolerance was initially set at $2\times 10^{-3}$, loose enough to miss the uncorrected jitter bias at 15° ($6.5\times 10^{-5}$); tightening it below the estimator’s own reproducibility of $4.9\times 10^{-6}$ would instead have made it fire on correct code.

These are recorded because they are the same class of failure the paper is about, occurring one level up: a validation apparatus can return plausible wrong answers exactly as a pipeline can. The regression tests accompanying this study therefore assert the two properties that make it meaningful—that no check fires on a correct pipeline, and that the self-consistency control never fires—rather than asserting the study’s conclusions.

### 3.7. The Same Chain on a Real Scanner

**Figure 5:**
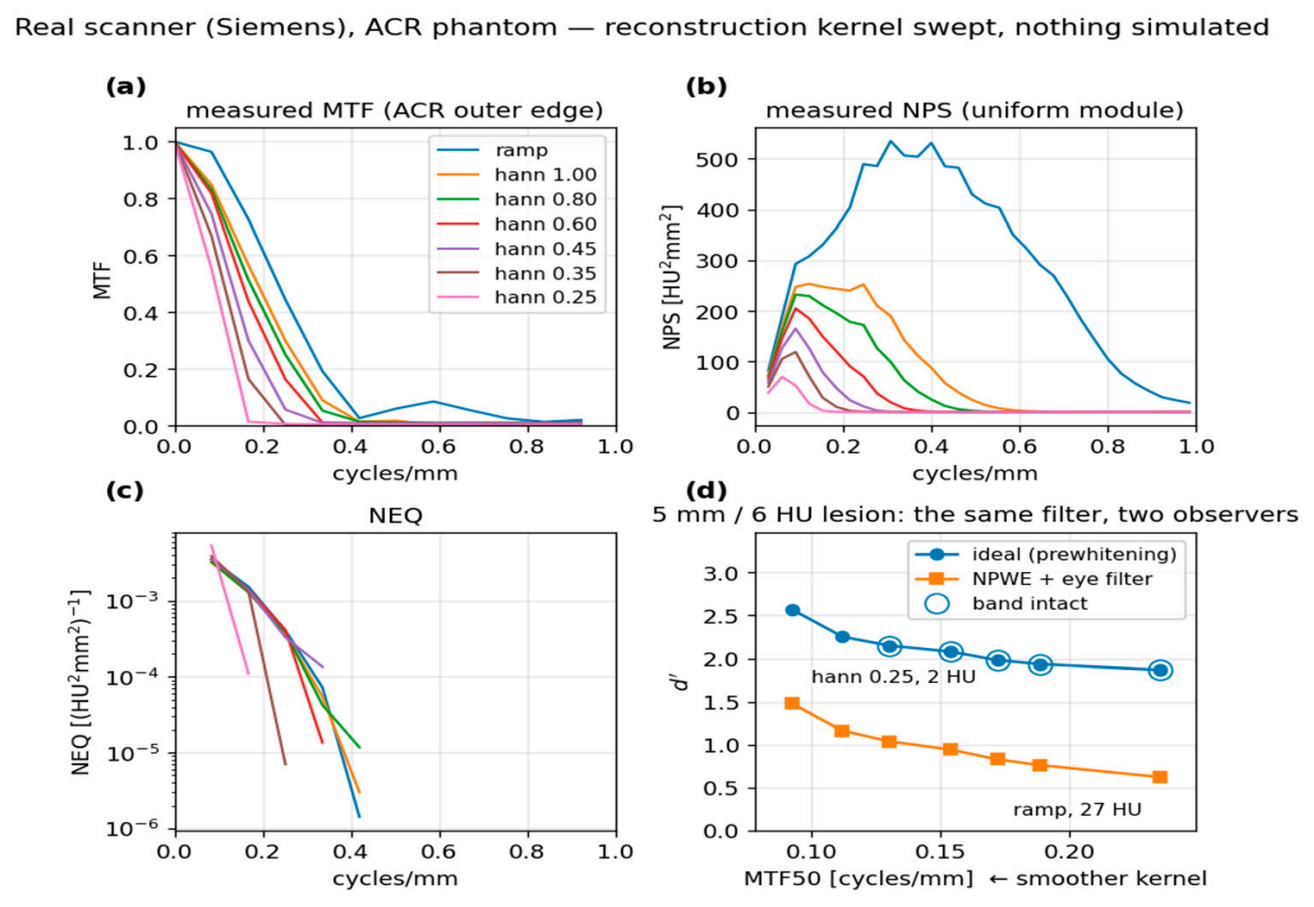
The same chain on measured ACR phantom projections, with the reconstruction kernel swept from a bare ramp through Hann apodisation at cutoffs 1.00 to 0.25. (**a**) measured MTF; (**b**) measured NPS; (**c**) NEQ; (**d**) detectability for the ideal and the non-prewhitening eye-filter observer against MTF_50_. Nothing about the acquisition is simulated; the same projections are reconstructed seven ways.

Run without modification on measured ACR phantom projections, the chain behaved as the theory requires across all seven reconstruction kernels (Table 3, Figure 5).

Strengthening the apodisation reduces resolution and noise together, as it must. That the two move together under a change in reconstruction is the ordinary behaviour of the chain, and it is measured the same way on newer detectors: a recent phantom study of ultra-high-resolution photon-counting CT reports noise texture and high-contrast resolution as the paired quantities that a change in pixel size moves [20], which is the same coupling seen here under a change in kernel. The checks in this paper are stated on those quantities rather than on any particular detector, and so they apply to that setting unchanged. Ideal-observer detectability rises monotonically from 1.87 to 2.57 across the sweep: for this low-contrast task, the noise reduction outweighs the resolution loss throughout the range tested. The efficiency of the non-prewhitening eye-filter observer relative to the ideal observer rises three-fold over the same sweep, from 0.111 to 0.331—the inefficient observer benefits from smoothing far more than the efficient one does, because smoothing performs part of the noise-weighting the inefficient observer cannot perform for itself.

The regression of ${d^{\prime }}_{\mathrm{ideal}}^{2}$ on the NEQ integral returns $R^{2}=0.84$ here, against a coefficient numerically equal to 1 on synthetic data. The gap is instructive: at the two strongest apodisations, the measured MTF band has collapsed to 0.25 and 0.17 mm^−1^, so the NEQ integral is taken over a truncated band and no longer summarises the same quantity. Over the five kernels whose measured band is intact, the NEQ integral varies by a coefficient of variation of 4.9% and $d_{\mathrm{ideal}}^{\prime }$ by 5.0%, while the noise standard deviation varies by a factor of 7.3.

### 3.8. Which Checks Survive the Move to Measured Data

Three of the four internal identities were run on all seven real-scanner reconstructions (Table 4). The fourth, bridge, is absent for a reason specific to measured data rather than by omission: it compares a prewhitening observer using the measured noise spectrum against an NEQ route that requires an analytic noise scalar, and on a physical scanner those two routes no longer share a noise model. What it then measures is that disagreement, not the correctness of the pipeline, and Section 3.6 gives the residual it produces. The claim of transfer in this paper is therefore made for three identities, not four.

Three things follow, and the third was not anticipated.

**Parseval survives intact.** The identity holds to $4.4\times 10^{-16}$—machine precision—on measured projections, exactly as on synthetic data. This is the property that makes internal identities worth having: nothing about the transition from a generated phantom to a physical one weakens them because they never depended on knowing the answer.

**Tolerances do not survive intact.** The signal-area residual is $4.7\times 10^{-3}$ throughout, four orders of magnitude above the $10^{-6}$ that the same identity achieves on synthetic data, and constant across every kernel—which identifies its source as the discretisation of the disc rather than anything the reconstruction does. An identity transfers to measured data; the tolerance it should be held to does not, and it must be re-derived from the discretisation actually in use.

**A clinically ordinary reconstruction comes within 0.6% of tripping the guard.** The NPS dynamic range rises monotonically with apodisation, from $3.1\times 10^{3}$ at a bare ramp to $9.9\times 10^{5}$ at Hann 0.25—against the $10^{6}$ threshold beyond which a prewhitening observer is refused. The strongest smoothing tested, which is not an exotic setting, leaves the spectrum a factor of 1.006 short of the point at which the software declines to compute a detectability at all. The margin protecting a real study from the $d^{\prime }\approx 10^{29}$ failure mode of Section 1.1 is therefore not comfortable, and it narrows in exactly the direction that low-dose protocols push reconstruction. This is a guard that will fire in practice, and an implementation without one will instead return a number.

## 4. Discussion

### 4.1. What This Implies for Reported Studies

The uncomfortable corollary of Section 3.3 is that a large fraction of published model-observer work carries no evidence bearing on the class of defect studied here. Reviews of task-based practice in CT describe the estimators and the observers in detail and say little about how an implementation of them is shown to be correct [9], and the wider evidence on software defects in published science suggests that silence is not because the problem is absent [12,13]. The usual reproducibility apparatus—a fixed seed, a pinned environment, a stored expected output, code released on request—establishes that a result can be regenerated. None of it establishes that the result was right the first time. A defect present at first publication is reproduced faithfully by every subsequent re-run, and released code makes the reproduction easier rather than the error more visible.

We do not claim that published detectability values are commonly wrong; we have not surveyed them, and this study cannot support such a claim. What it does support is narrower and still uncomfortable: for six defects of a kind that occur in practice, the standard apparatus provides zero detection power, and the errors they produce in a reported $d^{\prime }$ reach 90%.

### 4.2. A Checklist

Six checks, in two families. Nothing else in this list is a check.

**Family A—the four internal identities.** Cheap, needing no phantom, and assertable inside any implementation of this chain:

**A1.** ∫NPS(f) df equals the pixel variance of the data the NPS was estimated from, to floating-point tolerance.
**A2.** Ideal-observer $d^{\prime }$ computed through NEQ equals $d^{\prime }$ computed by the prewhitening observer, when both use the same noise model.
**A3.** The integral of a blurred signal equals the integral of the unblurred signal.
**A4.** The NPS dynamic range, excluding DC, stays within the range where 1/NPS is meaningful; a prewhitening observer should refuse rather than return a number when it does not. Section 3.8 shows this is not a theoretical precaution: strong apodisation on a real scanner approaches the threshold closely enough that the check decides real cases.

**Family B—the two closed-form references.** These require a phantom, and catch what the identities cannot:

**B1.** The presampled MTF of an analytically blurred edge equals $\exp\left(-2\pi^{2}\sigma^{2}f^{2}\right)$.
**B2.** The NPS of white noise of known variance equals $\sigma^{2}\;\Delta x\;\Delta y$

The injection study adds two methodological requirements to these six checks. Check **B1** must be evaluated over a *sweep* of edge angles, not one, because the bias it detects is not monotone in angle and can be an order of magnitude larger between two angles half a degree apart. Additionally, every tolerance must be set from the estimator’s measured reproducibility in the configuration actually in use: too loose and it misses the defect, too tight and it fires on correct code, and the correct value differs by four orders of magnitude between synthetic and measured data for the same identity.

### 4.3. Relation to Previous Work

Standards documents [3,4] and task-group reports [10] specify what to measure and how, and they are the appropriate reference for the definitions used here. They do not, and are not intended to, specify how an implementation should establish that it has implemented them correctly. Model-observer methodology reviews [7] treat the estimation of observer performance from data, largely under the assumption that the physical inputs are correct. The present study concerns the layer between: the correctness of the implementation itself, treated as an empirical question with a measurable answer.

The closest methodological relatives are outside imaging—mutation testing in software engineering, which measures a test suite by injecting faults and counting those it detects [21]. The adaptation here is that the “tests” are physical identities rather than assertions about programme state, and that the endpoint is a physical quantity ($d^{\prime }$) rather than test-suite coverage, so that detection can be dated against the point at which the science, not the code, goes wrong.

### 4.4. Limitations

The injected defects are six, chosen because three had actually occurred and three are standard; they are not a random sample of the space of possible defects, and the detection rates should not be read as an estimate of coverage against defects in general. The severity dial is a construction: real defects are present or absent rather than continuous, and severity here stands in for the free parameters (edge angle, bin width, ramp amplitude, correlation length) that determine how much a present defect costs. The material-error threshold of 5% is a convention.

The pipeline is a linear-systems idealisation: shift-invariant imaging, stationary noise, Gaussian blur, and a signal- and background-known-exactly task, which is the most tractable and least clinically realistic paradigm. The real-scanner arm demonstrates that the chain runs and behaves correctly on measured projections; it is not a validation against a physical standard, since the true MTF and NPS of that scanner are unknown—which is the point of Section 3.4 rather than an oversight. The channelised Hotelling observer has no closed form and is not part of the analytic sweep.

Finally, the study validates a pipeline against identities that the same author selected. An identity that no one thought to assert protects nothing, and the four here are certainly not exhaustive.

## 5. Conclusions

A task-based image quality pipeline that passes its own regression suite has demonstrated stability, not correctness, and for the class of defect studied here, the distinction is total: self-consistency caught none of the six injected defects, while closed-form and identity-based checks caught all six, in every case at or before the point where the reported detectability became materially wrong—which, for three of the six, it never did. The two families of checks are complementary and neither suffices alone. Half the defects are detectable using identities that need no ground truth and therefore travel to patient data; the other half are invisible without a phantom whose answer is known in closed form. A workflow that discards the phantom once it moves to real images does not lose a little sensitivity—it loses an entire class of error, permanently and without indication.

## Figures and Tables

**Table 1:** The six checks, their family, and the tolerance each is held to. Tolerances are set from the estimator’s measured reproducibility on a correct pipeline, with an order of magnitude of margin.

Key	Check	Family	Tolerance
parseval	∫NPS df = pixel variance	identity	$10^{-9}$
bridge	NEQ integral = prewhitening $d^{\prime }$	identity	$10^{-9}$
signal_area	signal integral = contrast $\times \pi r^{2}$	identity	$10^{-6}$
dynamic_range	$\max\left(\mathrm{NPS}\right)/\min\left(\mathrm{NPS}\right)<10^{6}$	identity	—
mtf_closed_form	MTF = $\exp\left(-2\pi^{2}\sigma^{2}f^{2}\right)$	closed form	$5\times 10^{-5}$
nps_closed_form	NPS = $\sigma^{2}\;\Delta x\;\Delta y$	closed form	0.25

**Table 2:** Six injected defects against seven checks. Detection severity is the smallest α at which a check fires; material severity is the smallest at which the reported $d^{\prime }$ is wrong by more than 5%. The seventh check, the self-consistency regression test, fired on none of the six and so appears nowhere in the *caught by* column. **Boldface in that column marks a check that is an internal identity**—one needing no phantom and no ground truth, and therefore still available on measured patient data. A defect whose entry carries no bold was caught only against an object whose answer was known in advance.

Defect	Origin	Caught by	Detection α	Material α	Worst Error in $d^{\prime }$
sinc	standard	MTF closed form	0.1	never	0.06%
jitter	found here	MTF closed form	0.1	never	0.002%
nps_area	standard	**Parseval**, NPS closed form	0.1	0.1	90.0%
detrend	standard	NPS closed form	0.1	0.2	69.4%
disk	found here	**signal area**	0.1	never	2.4%
no_floor	found here	**NPS dynamic range**, NPS closed form	0.1	0.1	37.0%

**Table 3:** MTF → NPS → NEQ → detectability on measured projections, sweeping apodisation. Nothing is simulated; the same projections are reconstructed seven ways.

Kernel	MTF_50_ (mm^−1^)	Noise SD	NEQ Peak	$d^{\prime }$ Ideal	NPWE Efficiency
ramp	0.235	27.34	0.00352	1.868	0.111
Hann 1.00	0.188	10.69	0.00320	1.935	0.155
Hann 0.80	0.172	8.12	0.00326	1.979	0.174
Hann 0.60	0.154	5.56	0.00355	2.078	0.205
Hann 0.45	0.130	3.75	0.00360	2.149	0.233
Hann 0.35	0.112	2.65	0.00389	2.252	0.266
Hann 0.25	0.092	1.61	0.00534	2.572	0.331

**Table 4:** Internal identities evaluated on measured ACR phantom data. Three of the four appear here. The two closed-form references cannot: neither the true MTF nor the true NPS of a clinical scanner is known analytically. The fourth identity, bridge, cannot either, because its two routes stop sharing a noise model once the noise is measured rather than specified (Section 3.6 and Section 3.8).

Kernel	Parseval Residual	NPS Dynamic Range	Signal-Area Residual
ramp	$4.4\times 10^{-16}$	$3.1\times 10^{3}$	$4.7\times 10^{-3}$
Hann 1.00	$2.2\times 10^{-16}$	$1.6\times 10^{4}$	$4.7\times 10^{-3}$
Hann 0.80	$4.4\times 10^{-16}$	$2.9\times 10^{4}$	$4.7\times 10^{-3}$
Hann 0.60	$4.4\times 10^{-16}$	$1.0\times 10^{5}$	$4.7\times 10^{-3}$
Hann 0.45	$2.2\times 10^{-16}$	2.9 × 10^5^	$4.7\times 10^{-3}$
Hann 0.35	$2.2\times 10^{-16}$	$6.6\times 10^{5}$	$4.7\times 10^{-3}$
Hann 0.25	$4.4\times 10^{-16}$	$9.9\times 10^{5}$	$4.7\times 10^{-3}$

## Data Availability

The estimator library behind every synthetic number here—the phantom generators and the physical and observer estimators, with their unit tests—is taskiq-core v0.4.0, archived on Zenodo (version DOI 10.5281/zenodo.21422924; concept DOI 10.5281/zenodo.21422923 resolves to the latest version). The scripts that run the studies reported here (paper/make_injection_study.py and paper/make_figures.py), their outputs, and the tests that check them were added to the repository after that release was tagged and are therefore not part of the Zenodo archive; they are openly available at <https://github.com/Institute-of-One/taskiq-core> (accessed on 9 September 2026) under the MIT licence. The revision the results reported here were verified against is 4c238362cd8720a7c6a2eddc98347dd400106bbd (<https://github.com/Institute-of-One/taskiq-core/tree/4c238362cd8720a7c6a2eddc98347dd400106bbd> (accessed on 9 September 2026)). The injection study reported in Table 2 and Figure 4 is run by paper/make_injection_study.py, which writes both paper/figures/fig4_injection.png and paper/figures/injection.json; the values quoted for that study in Table 2 and in the text were transcribed from that file. Figure 1, Figure 2 and Figure 3 are produced by paper/make_figures.py, which writes paper/figures/results.json. The real-scanner arm requires a second package, and neither reproduces it alone. Reading DICOM-CT-PD projections, restoring acquisition order from headers, single-slice rebinning and filtered backprojection with selectable apodisation are in ldct-io (<https://github.com/Institute-of-One/ldct-io> (accessed on 9 September 2026), MIT); the NPS, NEQ and observer estimators applied to those reconstructions are in taskiq-core. Table 3 and Table 4 and Figure 5 are written to results/acr_atlas.json by examples/acr_atlas.py in ldct-io, which requires both packages in one environment. The projections themselves are the ACR_Phantom series of LDCT-and-Projection-data [18], available from The Cancer Imaging Archive under CC BY 4.0 and not redistributed here; the path to a local copy is supplied through the LDCT_IO_DATA environment variable. Generative AI (Claude Opus 5, Anthropic) was used as a tool for code scaffolding, test drafting, figure generation and manuscript drafting, as disclosed in Section 2.8. The author is solely accountable for the content and independently verified every result. No AI system is an author. This disclosure follows ICMJE and COPE guidance.

## References

[1] Barrett H.H., Myers K.J. (2004). Foundations of Image Science.

[2] International Commission on Radiation Units and Measurements (1996). Medical Imaging—The Assessment of Image Quality. ICRU Report 54.

[3] (2017). Photography—Electronic Still Picture Imaging—Resolution and Spatial Frequency Responses.

[4] (2015). Medical Electrical Equipment—Characteristics of Digital X-Ray Imaging Devices—Part 1-1: Determination of the Detective Quantum Efficiency.

[5] Myers K.J., Barrett H.H. (1987). Addition of a channel mechanism to the ideal-observer model. J. Opt. Soc. Am. A.

[6] Barrett H.H., Yao J., Rolland J.P., Myers K.J. (1993). Model observers for assessment of image quality. Proc. Natl. Acad. Sci. USA.

[7] He X., Park S. (2013). Model observers in medical imaging research. Theranostics.

[8] Barrett H.H., Myers K.J., Hoeschen C., Kupinski M.A., Little M.P. (2015). Task-based measures of image quality and their relation to radiation dose and patient risk. Phys. Med. Biol..

[9] Verdun F.R., Racine D., Ott J.G., Tapiovaara M.J., Toroi P., Bochud F.O., Veldkamp W.J.H., Schegerer A., Bouwman R.W., Hernandez Giron I. (2015). Image quality in CT: From physical measurements to model observers. Phys. Medica.

[10] Samei E., Bakalyar D., Boedeker K., Brady S., Fan J., Leng S., Myers K.J., Popescu L.M., Giraldo J.C.R., Ranallo F. (2019). Performance evaluation of computed tomography systems: Summary of AAPM Task Group 233. Med. Phys..

[11] Peng R.D. (2011). Reproducible research in computational science. Science.

[12] Soergel D.A.W. (2015). Rampant software errors may undermine scientific results. F1000Research.

[13] Merali Z. (2010). Computational science: …Error. Nature.

[14] Claessen K., Hughes J. (2000). QuickCheck: A lightweight tool for random testing of Haskell programs. Proceedings of the Fifth ACM SIGPLAN International Conference on Functional Programming (ICFP).

[15] Chen T.Y., Kuo F.C., Liu H., Poon P., Towey D., Tse T.H., Zhou Z. (2018). Metamorphic testing: A review of challenges and opportunities. ACM Comput Surv..

[16] Oberkampf W.L., Roy C.J. (2010). Verification and Validation in Scientific Computing.

[17] Burgess A.E. (1994). Statistically defined backgrounds: Performance of a modified nonprewhitening observer model. J. Opt. Soc. Am. A.

[18] Moen T.R., Chen B., Holmes D.R., Duan X., Yu Z., Yu L., Leng S., McCollough C.H. (2021). Low-dose CT image and projection dataset. Med. Phys..

[19] Friedman S.N., Fung G.S.K., Siewerdsen J.H., Tsui B.M.W. (2013). A simple approach to measure computed tomography (CT) modulation transfer function (MTF) and noise-power spectrum (NPS) using the American College of Radiology (ACR) accreditation phantom. Med. Phys..

[20] Song K.-H., Shan C., Xu G., Duan X., Zhang Y., Zhang K., Ren L. (2026). Phantom evaluation of small-pixel effect in ultra-high-resolution photon-counting CT: Noise texture and high-contrast spatial resolution. J. Appl. Clin. Med. Phys..

[21] Jia Y., Harman M. (2011). An analysis and survey of the development of mutation testing. IEEE Trans. Softw. Eng..

